# Kavain Alleviates Choroidal Neovascularization Via Decreasing the Activity of the HIF-1α/VEGF-A/VEGFR2 Signaling Pathway and Inhibiting Inflammation

**DOI:** 10.34172/apb.2024.036

**Published:** 2024-03-11

**Authors:** Xi Chen, Xun Qin, Wen Bai, Junsong Ren, Yang Yu, Huiling Nie, Xiumiao Li, Zhangyu Liu, Jiayu Huang, Juxue Li, Jin Yao, Qin Jiang

**Affiliations:** ^1^The Affiliated Eye Hospital, Nanjing Medical University, Nanjing, Jiangsu 210029, China.; ^2^Department of Ophthalmology, Northern Jiangsu People’s Hospital, Yangzhou, 225001, China.; ^3^State Key Laboratory of Reproductive Medicine and Offspring Health, Nanjing Medical University, Nanjing, Jiangsu 211166, China.; ^4^Key Laboratory of Human Functional Genomics of Jiangsu Province, Nanjing Medical University, Nanjing, Jiangsu 211166, China.

**Keywords:** Neovascular age-related macular degeneration, Kavain, Anti-inflammation, Anti-angiogenesis, Choroidal neovascularization

## Abstract

**Purpose:**

Neovascular age-related macular degeneration (nAMD) is a prevalent cause of blindness in the elderly. Standard treatment includes anti-vascular endothelial growth factor (anti-VEGF) drugs, such as aflibercept. However, anti-VEGF drugs may have limited efficacy and cause drug resistance. This study explores whether Kavain, an anti-inflammatory molecule from Piper methysticum, can treat choroidal neovascularization (CNV).

**Methods:**

Various experiments were conducted to assess the Kavain’s toxicity. The impact of Kavain on in vitro cultured endothelial cells was examined through 5-ethynyl-20-deoxyuridine (EdU) assays, transwell migration assays, and tube formation assays. The therapeutic effects of Kavain on CNV were investigated using a laser-induced CNV mice model. To elucidate the mechanism of Kavain, network pharmacology analysis, molecular docking, and western blots were performed.

**Results:**

Kavain exhibited no apparent toxicity both in vitro and in vivo. Kavain significantly decreased endothelial cell viability, proliferation, migration, and tube formation ability in a dose-dependent manner compared to the hypoxia groups (*P*<0.05). Kavain alleviated CNV in the laser-induced CNV mouse model compared to the control groups (*P*<0.05). These effects were statistically significantly enhanced in the Kavain plus aflibercept groups (*P*<0.05). Following Kavain administration, the expression levels of various inflammatory factors were markedly reduced in retinal pigment epithelium (RPE)/choroid complexes (*P*<0.05). Mechanistically, Kavain decreased the activity of the hypoxia-inducible factor 1α (HIF-1α)/VEGF-A/ VEGF receptor 2 (VEGFR2) signaling pathway.

**Conclusion:**

Our study is the first to demonstrate Kavain’s potential as a promising treatment for nAMD, owing to its dual effects of anti-inflammation and anti-angiogenesis.

## Introduction

 Age-related macular degeneration (AMD) is a common ocular fundus disease that increases in prevalence with age.^1,2^ Neovascular age-related macular degeneration (nAMD) stands out as a leading cause of irreversible blindness worldwide.^3^ Choroidal neovascularization (CNV) represents the hallmark of nAMD. The abnormal choroidal blood vessels could lead to retinal or subretinal hemorrhage, macular edema and exudation, photoreceptor injury, fibrovascular scarring, and permanent vision loss if left untreated.^4,5^ Vascular endothelial growth factor A (VEGF) is a well-known potent growth factor in the development of ocular angiogenesis.^6^ Anti-VEGF therapy, including aflibercept, has been the gold standard for treating nAMD.^7^ However, anti-VEGF therapy faces various issues, such as drug resistance to post-intravitreal injections and lack of response in certain individuals.^8^ The pathogenesis of nAMD is complex and remains largely unknown. It is widely believed that hypoxia and inflammation contribute to the development and aggravation of nAMD.^4,9-13^ It has been established that a range of inflammation-related factors, including tumor necrosis factor (TNF-α), interleukin-6 (IL-6), and interleukin-1β (IL-1β), contribute to the progress of nAMD.^9,10,14,15^

 Subretinal fibrosis stands out as a severe complication in the advanced stage of nAMD, lacking viable treatment options.^4^ Within the context of subretinal fibrosis, a substantial influx of immune cells into the subretinal tissues ensues, initiating pronounced inflammation cascades.^4^ The sustained inflammatory response subsequently contributes to the emergence of macular fibrosis and the conversion of the neovascular membrane into a fibrovascular lesion.^16,17^ Consequently, recent emphasis in nAMD treatment has centered around anti-inflammatory drugs.^18^

 Kavain, a small molecule derived from the root of the Kava plant Piper methysticum, exhibits notable anti-inflammatory effects in various animal models.^19-21^ However, the impact of Kavain on the treatment of ocular diseases, particularly CNV, remains unexplored. In this study, we established a laser-induced CNV mouse model to investigate the influence of Kavain on CNV. Our experimental results demonstrate that Kavain significantly impedes the pathological angiogenesis associated with laser-induced CNV lesions, particularly when administered in conjunction with aflibercept. Kavain effectively diminishes the expression of multiple inflammatory factors. Furthermore, our study, for the first time, reveals that Kavain hinders angiogenesis, in part, by suppressing the activity of the hypoxia-inducible factor 1α (HIF-1α)/VEGF-A/VEGF receptor 2 (VEGFR2) signaling pathway. Collectively, our data underscore the anti-inflammatory and anti-angiogenic properties of Kavain in the context of nAMD, suggesting its potential therapeutic value in the treatment of this condition.

## Materials and Methods

###  Preparation of Kavain

 Kavain (MCE, HY-N2096, USA) was dissolved in dimethyl sulfoxide (DMSO; MCE, HY-Y0320, USA) to prepare a stock solution of the desired concentration.

###  Cell culture and hypoxia stimulation

 Human umbilical vein endothelial cells (HUVECs) were procured from the American Type Culture Collection (ATCC; USA). The HUVECs were cultured in Dulbecco’s Modified Eagle’s Medium (DMEM; Gibco, C11995, USA) supplemented with 10% fetal bovine serum (FBS; Gibco, 16140071, USA). The cells were maintained at 37 °C with a 5% CO_2_ atmosphere. HUVECs were subjected to either hypoxic conditions (1% O_2_) or normoxic conditions (20% O_2_).

###  Cell viability assay

 The cell viability of HUVECs was assessed using a Cell Counting Kit-8 (CCK-8) (Biosharp, BS350A, USA). HUVECs were seeded into 96-well plates at an equal density, and various treatments were applied once the cells had adhered completely. After different incubation times (24 h, 48 h, 72 h), the culture medium was removed, and 100 μL of a mixed CCK-8 solution (10% CCK-8 in DMEM) was added to each well. The plates were then incubated at 37 °C in the dark for 2 hours. Subsequently, a microplate reader measured the optical density (OD) at a wavelength of 450 nm.

###  Flow cytometry

 HUVECs were seeded in 6-well plates at an equal density. The cells were treated with different drugs for 24 hours after adhering completely to the plates. Cell apoptosis was identified utilizing an Annexin V-FITC/PI Apoptosis Detection Kit (Vazyme, A211-01, China). In brief, the cells were harvested after digestion with a 0.05% trypsin solution (EDTA-free), followed by resuspending in 100 μL binding buffer containing FITC Annexin V (5 μL) and PI (5 μL) in the dark for 10 minutes at room temperature. Data on cell apoptosis were collected and analyzed using a flow cytometer (BD Biosciences, USA) and FlowJo 7.6.5 software (FlowJo LLC, USA).

###  5-Ethynyl-20-deoxyuridine ( EdU ) assay

 A Beyo-Click^TM^ EdU Cell Proliferation Kit with Alexa Fluor 488 (Beyotime, C0071S, China) was employed to assess the proliferation of HUVECs. In brief, EdU reagents were introduced to HUVECs and incubated for 2 hours. Subsequently, cells were fixed, rendered permeable, and stained for 30 minutes using an azide dye solution. The cell nuclei were counter-stained with DAPI, and a fluorescence microscope captured images of the cells.

###  Transwell migration assay

 The migratory ability of HUVECs was assessed using a transwell migration assay. In summary, the lower chambers were filled with DMEM containing 10% FBS, while the upper chambers were seeded with HUVECs pre-treated with the drug for 24 hours (suspended in serum-free DMEM). The transwell assay was performed using transwell chambers with an 8.0 μm pore size (Millipore, USA). Following 12 hours of culture, cells were treated with methanol for 20 minutes and then stained with 0.3% crystal violet (C805211, Macklin, China) at room temperature for 20 minutes. Cell images were captured using an Olympus IX73 fluorescent microscope.

###  Tube formation assay

 The tube formation assay was conducted to assess the angiogenesis of HUVECs.^22^ An equal density of HUVECs (5 × 10^4^ cells per well), pretreated with drugs, was seeded in serum-free DMEM medium and cultured in a 24-well plate coated with growth-factor reduced matrigel (356230, Corning, USA) for 24 hours at 37 °C for 6 hours.^23^ The tube formation of HUVECs was observed and captured using a fluorescence microscope. The capacity for tube formation was analyzed using the Image J software program (http://rsb.info.nih.gov/ij/).

###  Animals

 C57BL/6J mice (males, 6 to 8 weeks old) were procured from the Nanjing Qinglongshan Animal Research Center (Nanjing, China). The mice underwent ophthalmic studies in accordance with the Association for Research in Vision and Ophthalmology (ARVO) Guidelines. Approval for all animal studies was obtained from the Institutional Animal Care and Use Committee of Nanjing Medical University (Nanjing, China). For surgical experiments, mice were anesthetized through intraperitoneal injection of ketamine (80 mg/kg) and xylazine (10 mg/kg). Subsequently, the animals were euthanized by cervical dislocation following an overdose of the anesthetic mixture.

###  Laser-induced CNV model

 After undergoing anesthesia and dilation of pupils, 8-week-old male C57BL/6J mice were subjected to laser photocoagulation treatment. Four laser burns around the optic nerve of the mouse were evenly induced, approximately two papillary diameters away from the optic nerve. The laser photocoagulation was performed using the following parameters (OcuLight GL, Iridex, Mountain View, CA): a wavelength of 532 nm, a duration of 0.1 s, power set at 120 mW, and a spot size of 50 µm. Following the laser photocoagulation procedure, the presence of a white subretinal bubble indicates the successful disruption of Bruch’s membrane.

###  Intravitreal injection

 The mice were randomly assigned to four groups, and each group received intravitreal injections of DMSO (vehicle control), Kavain, aflibercept, or Kavain plus aflibercept, respectively. Intravitreal injections were administered using an intravitreal Hamilton syringe equipped with a 30-gauge needle (Reno, USA). The mice underwent injections of 0.1% DMSO (Ctrl), Kavain (10 µg/mL), aflibercept (40 mg/mL), or a combination of Kavain and aflibercept into each eye, with a total volume of 2 µL. Following injection, an eye ointment containing levofloxacin hydrochloride was applied to the ocular surface. The mice were kept warm on a constant heat pad until fully awake.

###  Electroretinogram (ERG) measurement

 Before recording the ERG, mice were kept in complete darkness overnight. The pupils of the mice were dilated using topical 0.5% tropicamide and 2.5% phenylephrine after anesthesia. A contact electrode for mice was then placed on the central cornea of the tested eye after the mice were topically anesthetized with 0.4% oxybuprocaine hydrochloride eye drops. Steel needle electrodes for reference and ground were inserted into the skin of the cheeks, respectively. Flash ERG records were bandpass-filtered at 0.3 and 500 Hz and captured using the Color Dome LED/Xenon full-field stimulator (Diagnosys LLC, Lowell, MA). Measurements and recordings were taken for both the a-wave and b-wave amplitudes.

###  Intraocular pressure (IOP) measurement

 IOP measurements were conducted on mice utilizing a non-invasive tonometer (ICARE TONOLAB, Finland). The probe tip was positioned at a distance of 1 to 4 mm from the cornea’s surface, ensuring perpendicular contact with the center of the cornea. For each IOP reading, the tonometer probe made six consecutive contacts with the cornea’s center. The IOP was determined through an algorithm that relied on the probe’s incidence velocity and deceleration following six successful measurements. The resulting value was then exhibited on the display, presenting the IOP in mmHg.

###  Hematoxylin and eosin (HE) staining

 The HE staining procedure has been widely documented. Mouse tissues were fixed in 4% paraformaldehyde (PFA) for 24 hours at 4 °C. Subsequently, following dehydration with gradient alcohol, the tissues underwent embedding in paraffin, sectioning, deparaffinization, and sequential staining with hematoxylin and eosin. Photographs were captured using a fluorescence microscope (Olympus, IX73, Japan).

###  Terminal uridine nick-end labeling (TUNEL) staining

 The In Situ Cell Detection Kit (Beyotime, C1086, China) was utilized for the identification of apoptotic cells in retinal tissues via the TUNEL assay. Essentially, tissues underwent exposure to the TUNEL reaction liquids for 1 hour in darkness at 37 °C, following the guidelines provided by the kit. A pre-treatment with recombinant DNase I (Beyotime, C1082, China) was administered to the positive control sample. DAPI solutions (Beyotime, C1002, China) were then added to the sections for labeling nuclei before observation and photography using a fluorescence microscope.

###  Isolectin-B4/ immunofluorescence staining

 The eyeballs of the mice were enucleated and subsequently fixed for 30 minutes at room temperature in 4% PFA. Following fixation, the enucleated eyes were excised, and the corneas, lens, and retinas were carefully removed. Subsequently, the choroid was meticulously dissected into 4-8 petals. After a series of washing, blocking, and permeabilization steps, the retinal pigment epithelium (RPE)-choroid complexes were incubated with primary antibodies against Neutrophil, F4/80, and Ki67 (details of the antibodies could be seen in Table S1) at 4 °C overnight. This was succeeded by incubation with secondary antibodies, specifically Alexa Fluor 594 goat anti-rat IgG, combined with Alexa Fluor 488 Isolectin GS-IB4 (1:200, Invitrogen, 121411, USA) at room temperature for 4 hours, with protection from light. DAPI counter-staining was carried out on sections before capturing and recording the images with a fluorescence microscope, as required.

###  Enzyme-linked immunosorbent assay (ELISA)

 The RPE/choroid complexes of the mice were harvested, homogenized, and solubilized in radio-immunoprecipitation assay (RIPA) lysis buffer (Beyotime, P0013B, China) containing the protease inhibitor (Roche, Basel, Switzerland). Subsequently, the levels of monocyte chemoattractant protein-1 (MCP-1), IL-6, matrix metalloproteinase-9 (MMP9), and TNF-α were quantified using ELISA development kits (R&D Systems, Minneapolis, USA), following the manufacturer’s instructions. The OD was assessed at 450 nm using a microplate reader (Molecular Devices, FilterMax F5, USA).

###  Network pharmacology analysis

 To identify potential target genes, the term “choroidal neovascularization” was utilized as a keyword in the GeneCards database (https://www.genecards.org/). Genes with a Relevance Score > 2 were chosen as candidate genes. For predicting potential target genes of Kavain, the SuperPred database (https://prediction.charitede/), PharmMapper database (https://lilab-ecust.cn/), ChemMapper database (http://www.lilab-ecust.cn/chemmapper/), and SwissTargetPrediction database (https://swisstargetprediction.ch/) were employed. The overlapping genes from these databases were identified and subsequently input into the STRING database (https://string-db.org/) to generate the protein-protein interaction (PPI) network of “Homo sapiens”. The visualized PPI network was created using Cytoscape software (version 3.6.1). To further analyze the overlapped genes, Gene Ontology (GO) analysis and MSigDB_Hallmark analysis were conducted, and the results were obtained from the Enrichr web tool (https://maayanlab.cloud/Enrichr/). Bar graphs representing the outcomes were generated using the ggplot function from the R package-ggplot2.

###  Molecular docking

 The structure of the small molecule Kavain (PubChem CID: 5281565) was sourced from the PubChem database (https://pubchem.ncbi.nlm.nih.gov/). The three-dimensional structure of substrates and coordinates of Kavain were determined using the Ligprep module of Schrödinger software (https://www.schrodinger.com/). The Epik module was employed to identify all possible stereoisomers and associated protonation states of Kavain. To pinpoint the crystal structure of the specific protein, the Protein Data Bank (PDB) (https://www.ebi.ac.uk/pdbe/) was consulted. For the preparation of the protein structure and subsequent molecular docking, the Protein Preparation Wizard and Glide modules of Schrödinger software were utilized.

###  Western blot

 Proteins were extracted from cells or tissues by lysis in RIPA lysis buffer containing a protease inhibitor cocktail on ice. The quantification was performed using the bicinchoninic acid (BCA) Protein Assay (Bio-Rad, 23227, USA). Subsequently, an equal amount of protein samples (2 mg/mL, 10 µL) was transferred onto polyvinylidene fluoride (PVDF) membranes (Merck Millipore, Billerica, MA, USA). Following a 2-hour blocking step with 10% skim milk at room temperature, the PVDF membranes were incubated overnight at 4 °C with primary antibodies: HIF-1α, VEGF-A, phospho-VEGF receptor 2 (p-VEGFR2), VEGFR2, and β-actin (details of the antibodies could be seen in Table S1). Afterward, the PVDF membranes were subjected to a 2-hour incubation at room temperature with the appropriate dilution of secondary antibody. Protein bands were visualized using an enhanced chemiluminescence (ECL) detection kit (Beyotime, P0018S, China) and imaged with the Multi equipment (Tanon-5200, China).

###  Statistical analysis

 One-way analysis of variance (ANOVA) or the Kruskal-Wallis test, followed by Bonferroni’s post hoc test, was employed to compare multiple groups, assessing each group against every other group. The analysis and visualization were conducted using GraphPad Prism 9 software (GraphPad Software, Inc.). A *P* value < 0.05 was considered statistically significant. The data were presented as mean ± SEM.

## Results and Discussion

###  Kavain has no detectable cytotoxicity 

**Figure 1:**
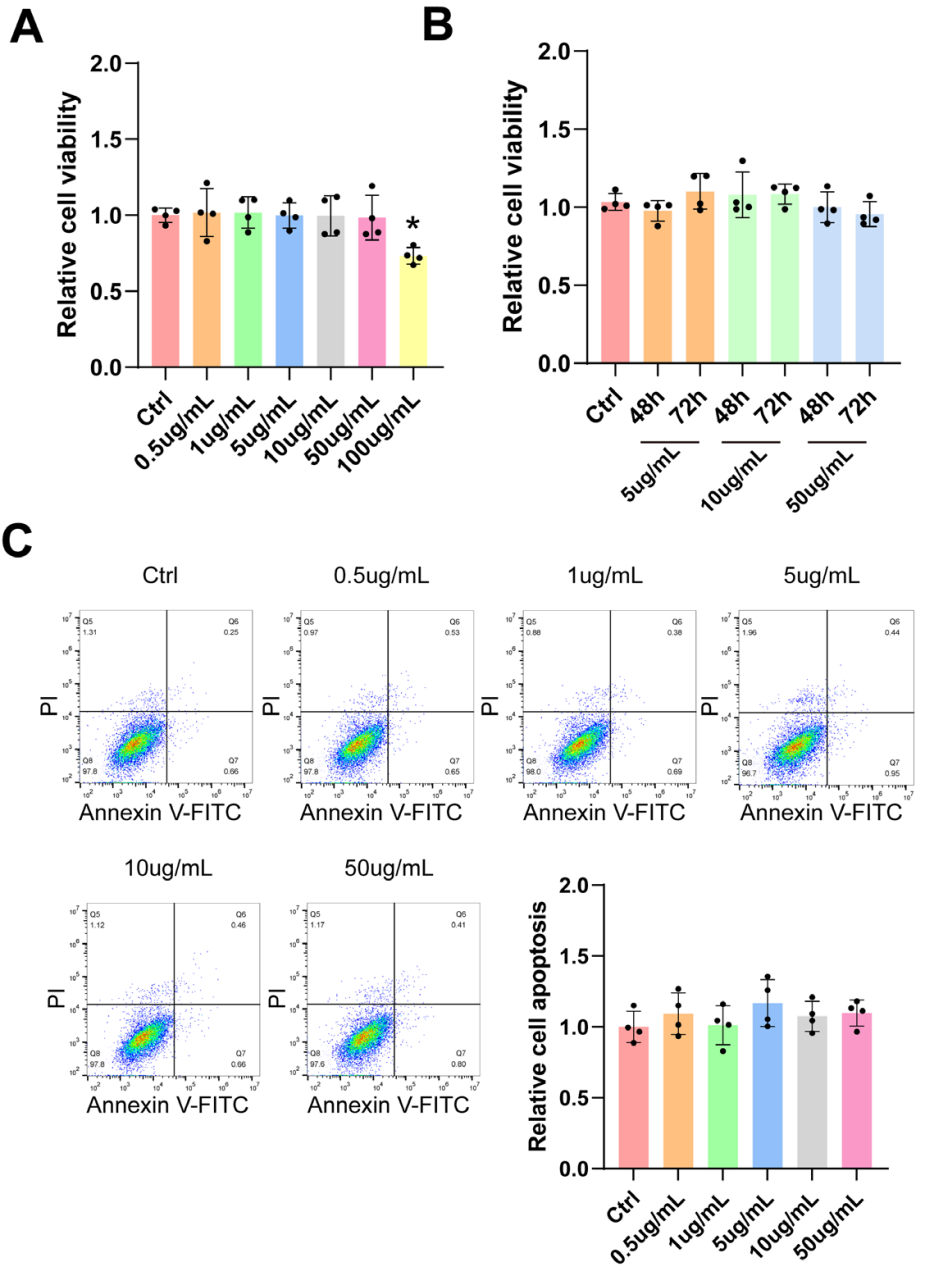
Kavain did not show obvious cytotoxicity toxicity at various concentrations in vitro. (A-C) HUVECs were incubated with different concentrations of Kavain (0.5 μg/mL-100 μg/mL) or left untreated (Ctrl) for 24 h (A, n = 4). HUVECs were incubated with Kavain (5 μg/mL-50 μg/mL) or left untreated (Ctrl) for 48 h or 72 h (B, n = 4). CCK-8 assays were conducted to detect cell viability (A and B). Representative panels of Annexin V-FITC/PI flow cytometry analysis and relative cell apoptosis of HUVECs cultured with different concentrations of Kavain (0-50 μg/mL) for 24 h (C, n = 4). A Bonferroni post hoc test followed one-way ANOVA. *P < 0.05 versus Ctrl group

 It is important to conduct *in vitro* toxicity tests to ascertain the appropriate drug concentration for functional verification. A CCK-8 assay was employed to assess the cytotoxicity of Kavain in HUVECs. The results of the CCK-8 assay indicated that Kavain did not induce cytotoxic effects within the concentration range of 0.5 μg/mL to 50 μg/mL (Figure 1A). Furthermore, concentrations of Kavain below 50 μg/mL exhibited no cytotoxicity in HUVECs over a 72-hour period (Figure 1B). Annexin/PI flow cytometry analysis failed to detect any noticeable apoptosis of HUVECs cultured with Kavain at concentrations below 50 μg/mL (Figure 1C).

 The results above revealed that Kavain did not exhibit apparent toxicity *in vitro*. Consequently, the toxicity assessment suggested that Kavain can be deemed a potential candidate for further experiments.

###  Kavain inhibits hypoxia-induced proliferation, migration, and tube formation in vitro

**Figure 2:**
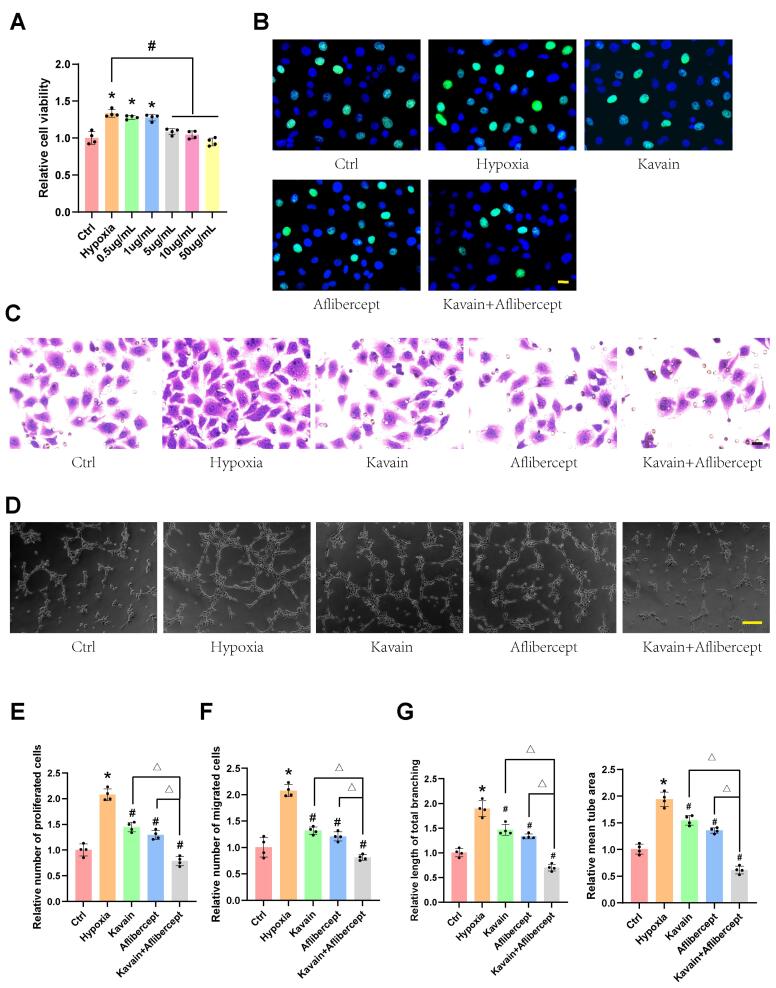
Kavain suppressed hypoxia-induced cell proliferation, migration, and tube formation *in vitro *in a dose-dependent manner. (A) HUVECs were cultured with hypoxia for 24 h, then treated with different concentrations of Kavain (0.5-50 μg/mL) for 24 h. CCK-8 assays were conducted to measure cell viability (A, n = 4). (B-D) HUVECs were pre-processed with hypoxia for 24 h, and then treated with Kavain (5 μg/mL), aflibercept (1 μg/mL), or Kavain (5 μg/mL) plus aflibercept (1 μg/mL) for another 24 h. Proliferation of HUVECs was determined by EdU assays (B, n = 4, bar = 20 μm). Transwell assays were conducted to migrate HUVECs (C, n = 4, bar = 20 μm). Representative images of HUVECs in Matrigel (D, n = 4, bar = 100 μm). Quantification of cell proliferation (E), migration (F), and tube formation (G). One-way ANOVA followed by Bonferroni post hoc test. *P < 0.05 versus Ctrl group. #*P* < 0.05 versus the hypoxia group. ^△^*P* < 0.05 Kavain or aflibercept versus Kavain + aflibercept group

 The anti-angiogenic potential of Kavain was investigated through CCK-8 assay, EdU assay, transwell migration assay, and tube formation assay in HUVECs. HUVECs were subjected to hypoxia for 24 hours, followed by treatment with various concentrations of Kavain (0.5-50 μg/mL) for an additional 24 hours (Figure 2A). Under hypoxic conditions, the cell viability of HUVECs significantly increased compared to normal oxygen conditions (Figure 2A). However, HUVECs treated with Kavain at concentrations ≥ 5 μg/mL exhibited significantly lower cell viability than the hypoxia groups (Figure 2A). Based on the CCK-8 assay results, Kavain at a concentration of 5 μg/mL was chosen for subsequent experiments.

 In further experiments, HUVECs were pre-conditioned with hypoxia for 24 hours and then treated with Kavain (5 μg/mL), aflibercept (1 μg/mL), or a combination of Kavain (5 μg/mL) and aflibercept (1 μg/mL) for an additional 24 hours (Figures 2B-D). EdU assays demonstrated that Kavain suppressed hypoxia-induced HUVECs proliferation, and the combination of Kavain and aflibercept enhanced this effect (Figures 2B and E). The transwell migration assay revealed that Kavain suppressed hypoxia-induced HUVECs migration, and the combination with aflibercept intensified this effect (Figures 2C and F). Additionally, Kavain administration reduced the hypoxia-induced tube formation ability of HUVECs, with the combination of Kavain and aflibercept displaying a stronger anti-tube formation ability (Figures 2D and G). These findings suggest that Kavain inhibits hypoxia-induced angiogenic effects in HUVECs* in vitro* in a dose-dependent manner.

###  Kavain has no detectable tissue toxicity

**Figure 3:**
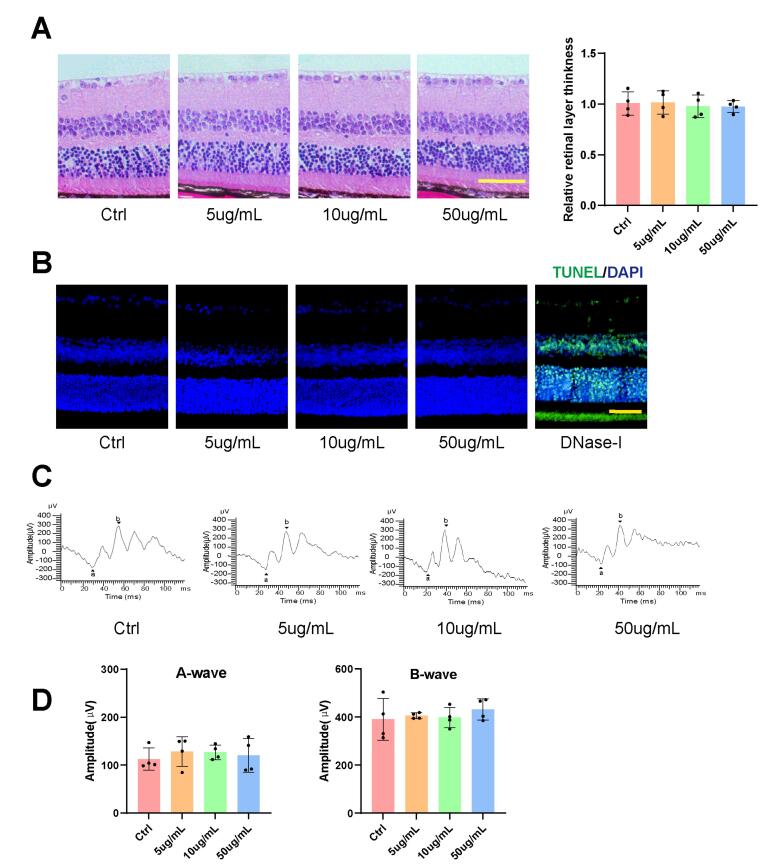
Kavain did not show obvious tissue toxicity at various concentrations *in vivo*. Representative HE staining images (left) of retina sections and statistical diagram (right) of retinal layer thickness (A, n = 4, scale bar = 50 μm). Representative TUNEL staining images (B, n = 4, scale bar = 50μm). Representative images of post-intravitreal Kavain ERG (C) and A-waves and B-waves amplitudes were analyzed statistically (D, n = 4). *P < 0.05

 The administration of Kavain* in vivo* was conducted to assess tissue toxicity. HE retina staining revealed no discernible morphological changes in the Kavain-treated groups across different concentrations (0-50 μg/mL) (Figure 3A). TUNEL assay did not identify a significant increase in apoptotic cells in retinal tissues following intravitreal injections of varying Kavain concentrations (0-50 μg/mL) (Figure 3B). Electroretinogram (ERG) testing showed no noteworthy alterations in either A-wave or B-wave amplitudes after the administration of different concentrations of Kavain (0-50 μg/mL) (Figures 3C and 3D). ERG examination indicated no apparent changes in retinal function following intravitreal injections of Kavain at concentrations less than 50 μg/mL when compared to the control group.

 Previous studies have suggested that intravitreal injection of drugs generally entails minimal systemic toxicity.^24-26^ However, in light of reports indicating potential liver damage caused by high doses of Kavain, we conducted liver histopathological examinations and hematological tests.^27^ The analysis of hematological parameters revealed no significant differences between the control group and treatment groups at various concentrations of Kavain, all less than 50 μg/mL (Figure S1A). HE staining sections of the livers exhibited no apparent morphological distinctions among the Kavain-treated groups spanning concentrations from 0 to 50 μg/mL (Figure S1B, Supplementary file 1).

 These findings indicate that Kavain demonstrated no apparent toxicity *in vivo*. Consequently, we opted for Kavain at a concentration of 10 μg/mL for subsequent *in vivo* experiments.

###  Kavain inhibits CNV in vivo

 We established a laser-induced CNV mouse model to evaluate the effects of Kavain on CNV. Previous investigations into nAMD have extensively utilized the mouse model featuring laser-induced CNV.^28,29^ This *in vivo* model relies on laser-induced damage to penetrate Bruch’s membrane, prompting the choroid to recruit blood vessels to the subretinal space.^28^ Intravitreal injections, each containing a volume of 2 μL, were administered to individual eyes of C57BL/6 mice one day after laser photocoagulation treatment. The injected substances included 0.1% DMSO (Ctrl), Kavain (10 μg/mL), aflibercept (40 mg/mL), or a combination of Kavain (10 μg/mL) and aflibercept (40 mg/mL). RPE-choroid-sclera complexes were then prepared for immunofluorescence staining 14 days after laser photocoagulation treatment.

**Figure 4:**
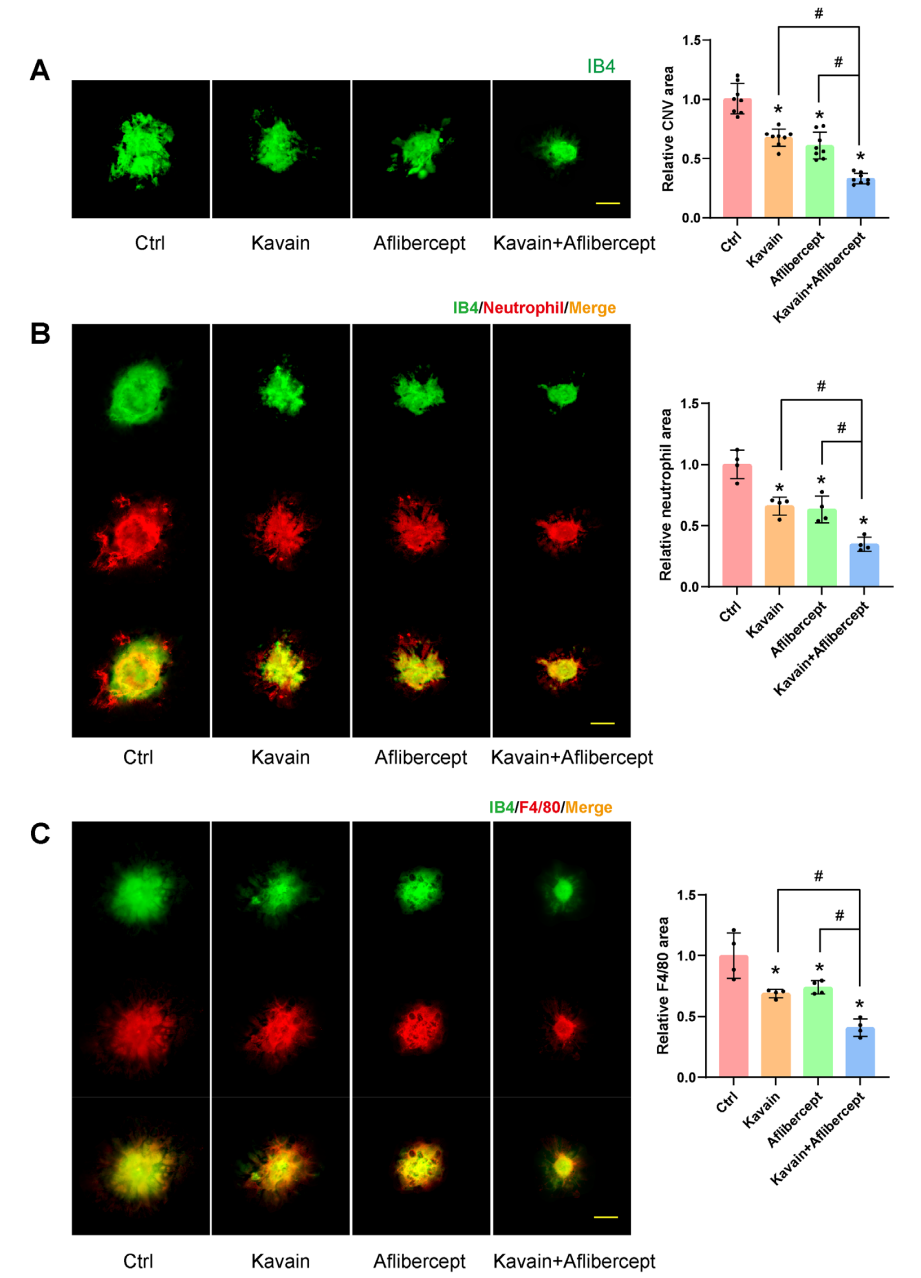
Kavain inhibited laser-induced CNV* in vivo*. (A-C) C57BL/6 mice received 2ul/eye intravitreal injections of 0.1% DMSO (Ctrl), Kavain (10 μg/mL), aflibercept (40 mg/mL), or a combination of Kavain (10 μg/mL) and aflibercept (40 mg/mL) one day after laser photocoagulation treatment. RPE-choroid-sclera complexes were prepared for immunofluorescence staining 14 days post-laser photocoagulation treatment. The CNV lesions were evaluated by IB4 (green) staining (A, n = 8). Neutrophil infiltration was quantified by immunofluorescence staining of the choroidal flat mounts with anti-Neutrophil (red) and IB4 (green) (B, n = 4). Macrophage numbers were quantified by immunofluorescence staining of the choroidal flat mounts with anti-F4/80 (red) and IB4 (green) (C, n = 4). Scale bar = 50 μm. * *P* < 0.05 versus Ctrl group. # *P* < 0.05 versus Kavain + aflibercept group

 IB4 staining was employed to detect CNV formation 14 days after laser-induced damage. The area of CNV lesions exhibited a significant reduction in both the Kavain-treated and aflibercept-treated groups compared to the laser-injured (Ctrl) group (Figure 4A). Furthermore, in comparison to both the Kavain-treated group and the aflibercept-treated group, the Kavain plus aflibercept group demonstrated minimal CNV lesions (Figure 4A).

**Figure 5:**
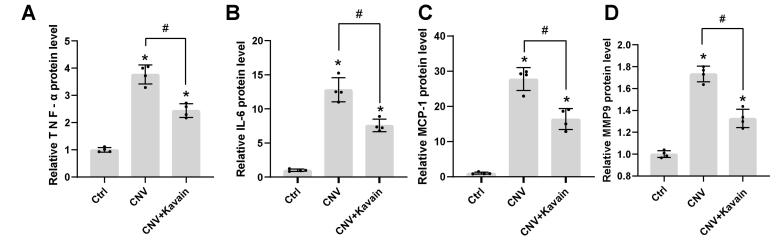
Kavain reduced the expression of different related inflammatory factors in the RPE/choroid complexes of CNV mice. Treat the CNV mice with Kavain intravitreally post-laser photocoagulation; untreated mice were taken as the control (Ctrl) group (n = 4). ELISA assays detected the relative protein expression level of TNF-α (A), IL-6 (B), MCP-1 (C), and MMP9 (D). N = 4. * *P* < 0.05 versus Ctrl group. # *P* < 0.05 versus CNV group

 We conducted Neutrophil and F4/80 staining on CNV to evaluate the impact of Kavain on inflammation during CNV. The Neutrophil and IB4 double staining revealed that the Kavain-treated group exhibited a smaller area of fluorescence compared to the control group (Figure 4B). In contrast to the laser-injured (Ctrl) group, the Kavain-treated group displayed a reduced fluorescence region, as evidenced by F4/80 and IB4 double staining (Figure 4C). The Kavain plus aflibercept group demonstrated the smallest Neutrophil and F4/80 staining area in comparison to the other groups (Figures 4B and 4C). Additionally, we utilized ELISA to determine the levels of inflammatory factors in RPE/choroid complexes. CNV mice were intravitreally treated with Kavain (10 μg/mL, 2 μL/eye) one day after laser photocoagulation, while untreated mice served as the control (Ctrl) group. RPE/choroid complexes were harvested three days post-photocoagulation. The levels of MMP9, MCP-1, IL-6, and TNF-α in RPE/choroid complexes of Kavain-treated groups were significantly lower than those of the control group (Figure 5). In brief, these assays demonstrated that Kavain mitigated inflammation in RPE/choroid complexes of laser-induced CNV mice.

**Figure 6:**
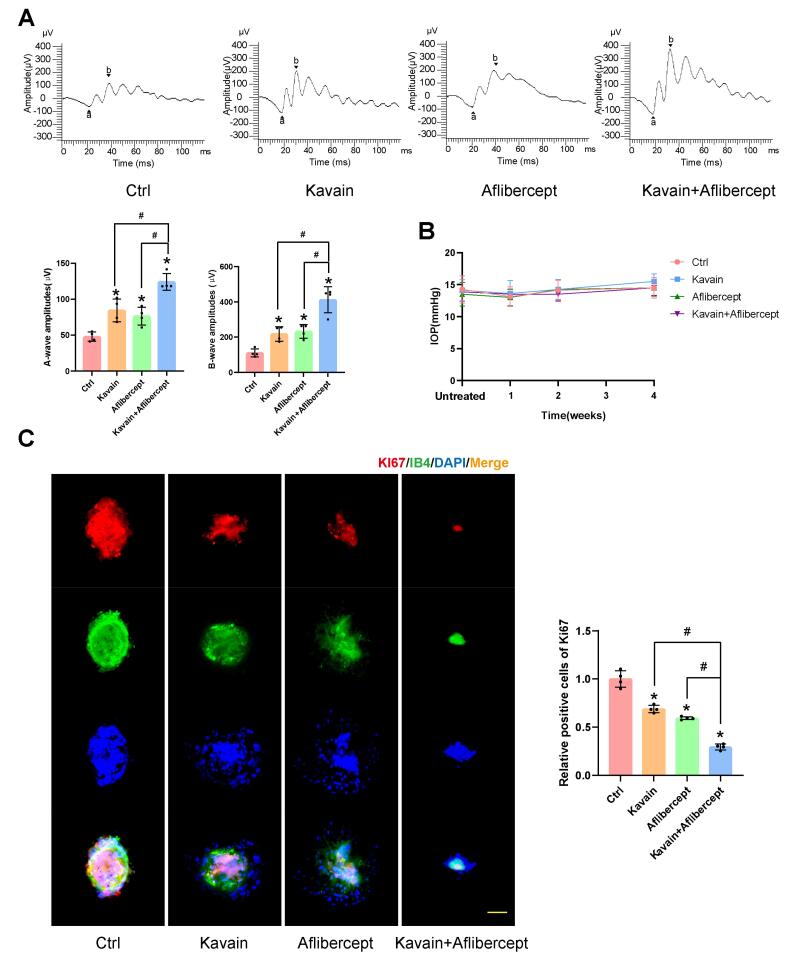
Kavain alleviated injury in the laser-induced CNV model. (A-C) The mice received 2 μl/eye intravitreal injections of 0.1% DMSO (Ctrl), Kavain (10 μg/mL), aflibercept (40 mg/mL), or a combination of Kavain (10 μg/mL) and aflibercept (40 mg/mL) one day after laser photocoagulation treatment. Representative ERG post-laser photocoagulation (A, n = 4). Statistical analysis results of IOP at different time points before or after intravitreal injection (B, n = 8). Represented images (left) of immunofluorescence staining of the choroidal flat mounts with anti-Ki67 (red), IB4 (green), and DAPI (blue), and quantification of the relative Ki67-positive cells (right) (C, n = 4, scale bar = 50 μm). * *P* < 0.05 versus Ctrl group. # *P* < 0.05 versus Kavain + aflibercept group

 ERG experiments were conducted to evaluate post-laser retinal function. Intravitreal injections, consisting of a 2 μL volume of 0.1% DMSO (Ctrl), Kavain (10 μg/mL), aflibercept (40 mg/mL), or a combination of Kavain (10 μg/mL) and aflibercept (40 mg/mL), were administered to the individual eyes of mice one day after laser photocoagulation treatment. Representative ERG images for each group, taken one month after laser photocoagulation treatment, are presented in Figure 6A. As anticipated, mice with laser-induced injuries exhibited diminished A-wave and B-wave amplitudes. However, both A-wave and B-wave amplitudes were significantly enhanced in the Kavain-treated and aflibercept-treated groups when compared to the control group. Furthermore, the Kavain plus aflibercept group exhibited the highest A-wave and B-wave amplitudes compared to the other groups.

 These findings demonstrated that Kavain possesses the ability to enhance retinal function following laser injury. IOP measurements taken before, at 1 week, 2 weeks, and 4 weeks after intravitreal injection of Kavain revealed no significant differences among the various time points (Figure 6B). This suggests that intravitreal injection of different drugs did not lead to a notable change in IOP. To evaluate the impact of Kavain on cell proliferation in laser-induced CNV, we conducted a proliferation-related immunofluorescence staining assay (Figure 6C). Both the Kavain-treated and aflibercept-treated groups exhibited significantly fewer Ki67 and IB-4 double-positive cells compared to the control group. Furthermore, the Kavain plus aflibercept group demonstrated the lowest number of Ki67 and IB-4 double-positive cells among all groups. Consequently, Kavain exhibited anti-cell proliferation capabilities in laser-induced CNV.

 Our *in vivo* experiments confirmed that Kavain reduces CNV through dual effects, specifically anti-angiogenesis and anti-inflammation.

###  Kavain inhibits CNV via reducing the activity of HIF-1 α/VEGF-A/VEGFR2 signaling pathway

**Figure 7:**
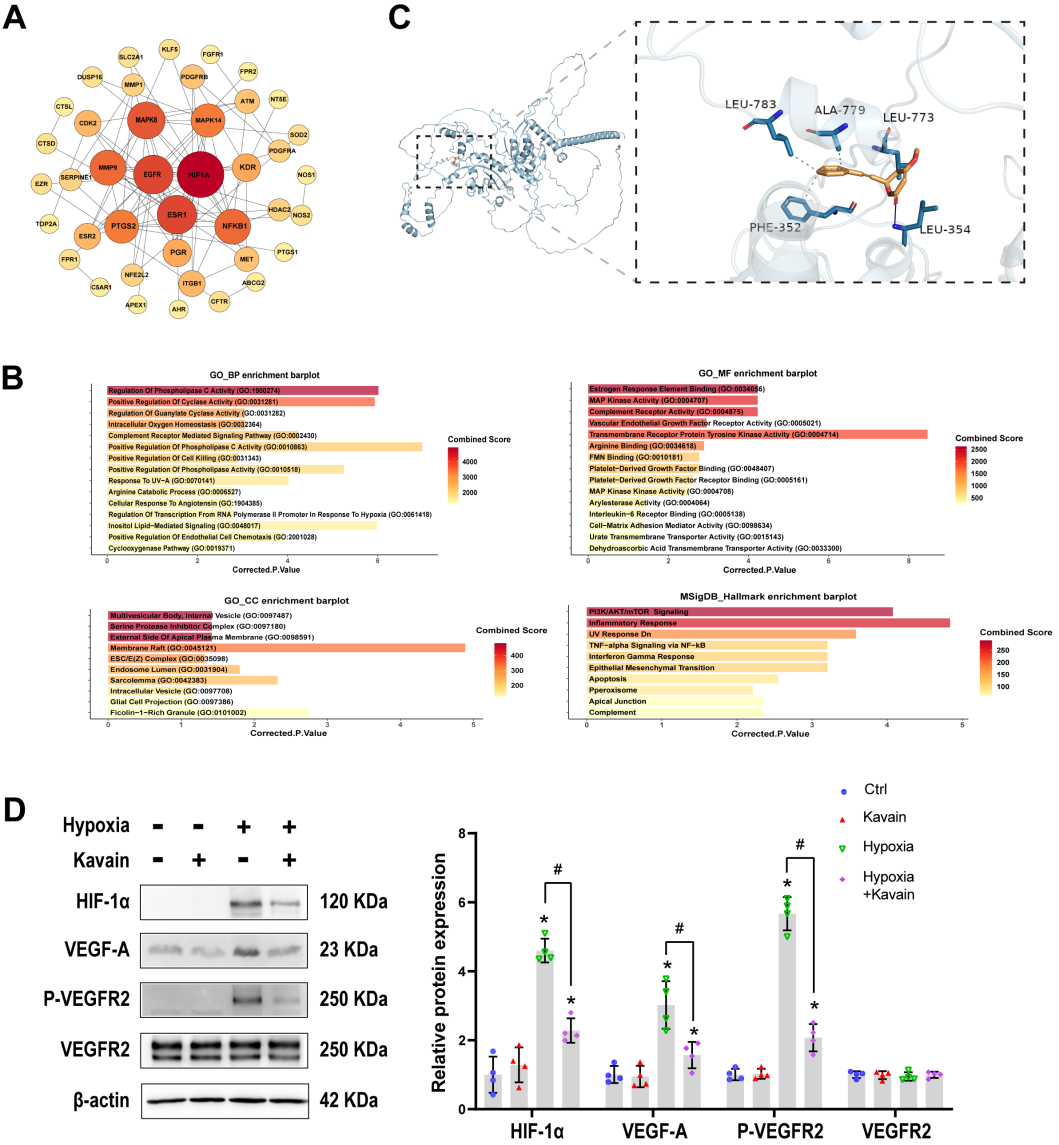
Kavain inhibits CNV related to HIF-1 α/VEGF-A/VEGFR2 signaling pathway. (A) PPI network analysis of target proteins. (B) GO enrichment and Hallmark enrichment analysis. (C) Molecular docking of Kavain with HIF-1 α. (D) HUVECs were pre-processed with or without hypoxia for 24 h, then treated with or without Kavain (5 ug/mL) for another 24 h. Representative western blot results and the quantitative data (n = 4). * *P* < 0.05 versus Ctrl group. # *P* < 0.05 versus the hypoxia group

 In the Genecards database, the term “choroidal neovascularization” was utilized as a keyword to search for genes associated with this process, and only the most relevant genes were selected. A total of 879 genes with a score ≥ 2 were identified. Additionally, 242 target genes of Kavain were sourced from the SuperPred database, Swiss target prediction database, PharmMapper database, and ChemMapper database. In the context of CNV, 47 overlapping genes were recognized as potential Kavain target genes (Figure 7A). The top 10 target genes identified were HIF-1α, EGFR, ESR1, MAPK8, MMP9, NFΚB1, PTGS2, MAPK14, KDR, and PGR. HIF-1α claimed the top position based on the PPI node. For a more comprehensive understanding, GO enrichment analysis was conducted, covering biological processes (BP), molecular functions (MF), and cellular components (CC), with results presented based on P values and combined scores (Figure 7B). Hallmark enrichment analysis was also provided, considering the P value and combined score (Figure 7B). Notably, intracellular oxygen homeostasis exhibited high significance in the BP enrichment barplot. To further substantiate these findings, a molecular docking investigation was conducted to assess the interaction between Kavain and HIF-1α, confirming a substantial binding affinity with binding energies of −8.7 kcal/mol (Figure 7C). Consequently, based on the network analyses conducted, HIF-1α emerges as the potential target for Kavain in the context of CNV.

 We conducted a Western blot to validate the results of the network analysis. HUVECs were pre-processed with or without hypoxia for 24 hours. Subsequently, they either received kavain treatment (5 μg/mL) or not for an additional 24 hours. The Western blots revealed a significant increase in the levels of HIF-1α, VEGF-A, and phosphorylation of VEGF Receptor2 in response to hypoxia stimulation in HUVECs. This indicates that hypoxia led to the activation of the HIF-1α/VEGF-A/VEGFR2 signaling pathway (Figure 7D). However, administration of kavain significantly decreased the protein levels of HIF-1α, along with the downstream signaling proteins VEGF-A and VEGF Receptor2 (Figure 7D). These results suggest that the anti-angiogenic effect of kavain was partially achieved through the suppression of the HIF-1α/VEGF-A/VEGFR2 signaling pathway.

 Kavain exhibited no toxicity at concentrations below 50 μg/mL, both *in vitro* and *in vivo*. It demonstrated the ability to inhibit hypoxia-induced proliferation, migration, and tube formation in HUVECs *in vitro*. Our laser-induced CNV mouse model showcased typical nAMD characteristics,^30,31^ with photocoagulation used to damage Bruch’s membrane and stimulate the formation of subretinal new choroidal vessels.^32^ Upon intravitreal injection, Kavain significantly inhibited CNV, and its combination with aflibercept demonstrated a more pronounced effect than that observed with Kavain or aflibercept alone. Previous *in vitro* and *in vivo* studies on Kavain have indicated its anti-inflammatory capabilities through various processes, particularly in suppressing TNF-α.^20,33,34^ Neutrophil or F4/80 staining of CNV and ELISA tests measuring the levels of inflammatory factors in RPE/choroid complexes confirmed the anti-inflammatory effect of Kavain in laser-induced CNV mice. Kavain was found to alleviate CNV by reducing the inflammatory response. Network pharmacological analysis was conducted to investigate the underlying therapeutic mechanism of Kavain in treating nAMD. Through PPI, GO enrichment analysis, and Hallmark analysis, we hypothesized that Kavain targets the HIF-1 α gene in CNV. Molecular docking analysis further affirmed the strong binding affinity between Kavain and HIF-1 α. Western blot assays subsequently validated that, under hypoxic conditions, Kavain inhibits the activity of the HIF-1 α/VEGF-A/VEGFR2 signaling pathway. Hypoxia-inducible factor (HIF), in conjunction with its downstream factors VEGF and VEGFR2, plays a pivotal role in the regulatory pathways of angiogenesis.^12,35^ Notably, VEGF is widely recognized as a key factor in both human nAMD and animal models of laser-induced CNV.^11^ Previous research has indicated that HIF binds to the hypoxia response element within the VEGF promoter region, activating the transcription of the VEGF gene.^36^ Intriguingly, our network pharmacological analysis revealed the potential interaction between Kavain and HIF-1 α. To validate this finding, we induced the activation of the HIF-1 α/VEGF-A/VEGFR2 signaling pathway under hypoxia conditions. Simultaneously, administration of Kavain led to a reduction in the expression of HIF-1 α, as well as its downstream targets VEGF-A and p-VEGFR2. To our knowledge, our research is the first to report the anti-angiogenic effects of Kavain.

 Angiogenesis and immunological dysfunction play crucial roles in the pathogenesis of nAMD, despite its complex etiology.^3,10^ Over the past few decades, anti-VEGF drugs, such as Bevacizumab, Ranibizumab, and aflibercept, have been widely employed to treat neovascular ocular diseases. These drugs exert their anti-angiogenic effects by binding to VEGF.^37^ Although anti-VEGF drugs have proven effective in treating nAMD, approximately 10%–15% of patients do not respond to the treatment.^38^ Inflammation is believed to be implicated in the development of resistance to anti-VEGF drugs in nAMD.^39^ Moreover, inflammation exacerbates the progression of nAMD.^40^ For instance, a persistent inflammatory response can lead to the formation of a fibrovascular lesion.^17^ Some scholars posit that hypoxia and HIF are central pathways in the pathogenesis of nAMD.^11,12,41^ However, no anti-inflammatory drug compares to anti-VEGF medicines alone in the real-world clinical treatment of nAMD.^42^ Therefore, the current research direction of nAMD treatment has shifted towards anti-inflammatory and non-anti-VEGF pathways.^9,10,43-46^ Our study demonstrates that Kavain suppresses CNV by alleviating inflammation and inhibiting angiogenesis. Additionally, the administration of Kavain plus aflibercept exhibited a more pronounced treatment effect than that of Kavain or aflibercept alone. The superior combination effect of Kavain plus aflibercept may be attributed to the dual effects of Kavain on CNV. Kavain’s anti-inflammatory and HIF-1α inhibition effects on CNV differ from those of regular anti-VEGF drugs, potentially making it a candidate drug for individuals resistant or non-responsive to anti-VEGF drugs. Notably, Kavain has not been studied in the eye. Furthermore, Kavain has not been reported to have the effect of inhibiting angiogenesis, and to our knowledge, this is the first report of its therapeutic efficacy in CNV treatment. As a small molecule drug, Kavain could be intravitreally injected at a high concentration in a relatively low volume. Intravitreal injection could also minimize the side effects of Kavain compared with systemic administration. No significant toxicity of Kavain was observed in our study, both *in vitro* and *in vivo*.

## Conclusion

 In conclusion, Kavain has been identified as having the unique ability to inhibit angiogenesis, marking a significant breakthrough. This study is the first to showcase Kavain’s inhibitory impact on CNV through its dual action as an anti-inflammatory agent and a suppressor of the hypoxia-induced HIF-1 α/VEGF-A/VEGFR2 pathway. Notably, the combined administration of Kavain and aflibercept yielded a more pronounced treatment effect compared to the use of Kavain or aflibercept alone. Therefore, Kavain emerges as a promising candidate for clinical treatment of nAMD. Despite these findings, the precise mechanism underlying Kavain’s efficacy in nAMD treatment remains unclear and warrants further investigation. Additionally, exploring the pharmacokinetics of Kavain following intravitreal injection represents a prospective avenue for future research.

## Acknowledgments

 This study is supported by the National Natural Science Foundation of China grants 82070983 and 82271101(to Q.J), and 82271107 (to J.Y). We also thank Figdraw (www.figdraw.com) for their help in creating the images.

## Competing Interests

 The authors declare no conflict of interest.

## Ethical Approval

 Not applicable.

## Supplementary Files

Supplementary file 1 contains Figure S1 and Table S1.
