# Supplementary material for: Kavain Alleviates Choroidal Neovascularization Via Decreasing the Activity of the HIF-1α/VEGF-A/VEGFR2 Signaling Pathway and Inhibiting Inflammation
Source: Adv Pharm Bull. 2024 Mar 11;14(2):469–82. doi: 10.34172/apb.2024.036 (PMC11347728; doi:10.34172/apb.2024.036)
Supplement: Supplementary file 1 — contains Figure S1 and Table S1. [file apb-14-469-s001.pdf]

Adv Pharm Bull, 2024, 14(2), 469-S1

doi: 10.34172/apb.2024.036

https://apb.tbzmed.ac.ir

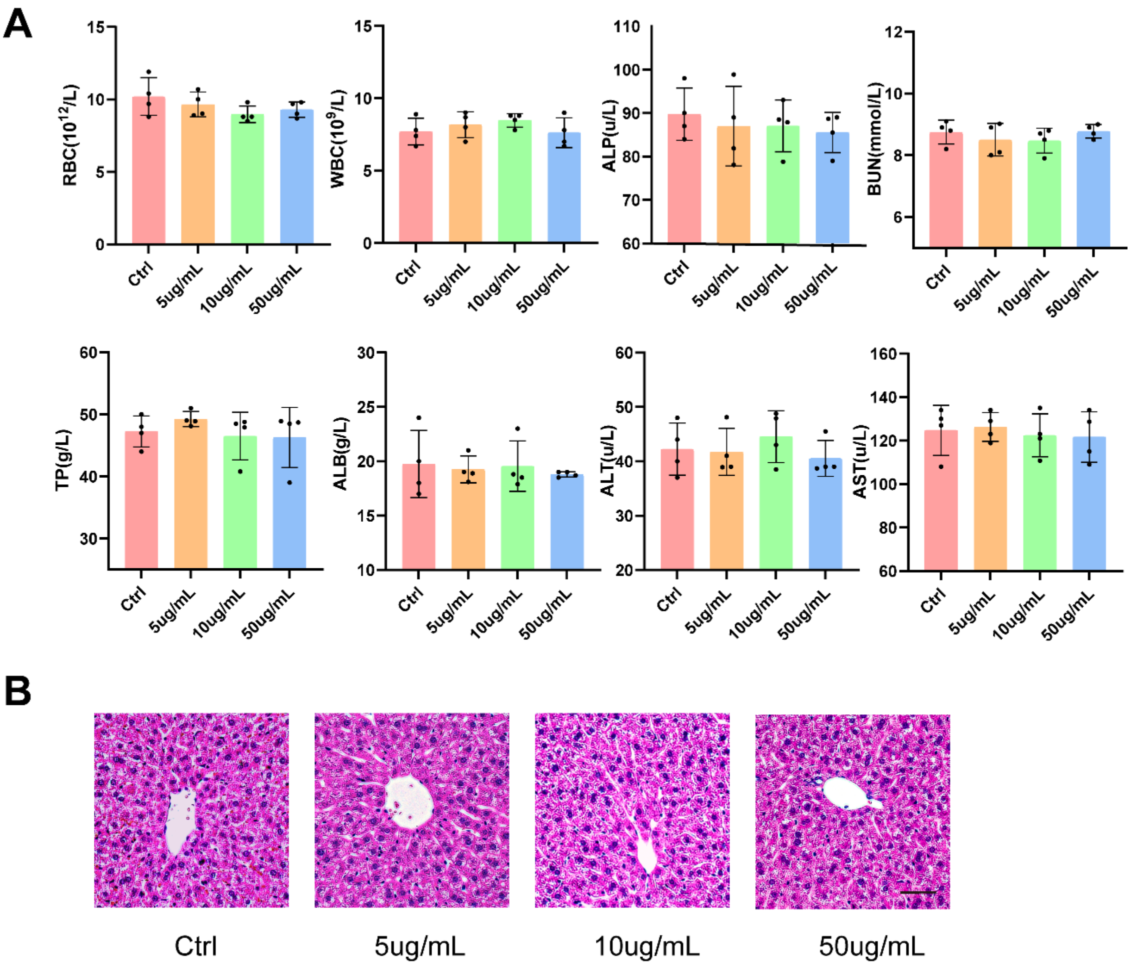

Figure S. 1. Toxicity of Kavain on male mice. (A) Analysis of chemistry parameters (*i.e.*, red blood cell [RBC], white blood cell [WBC], alkaline phosphatase [ALP], blood urea nitrogen [BUN], total protein [TP], albumin [ALB], alanine aminotransferase [ALT], aspartate aminotransferase [AST]) of male mice intravitreal injected with Kavain or DMSO (Ctrl) for 1 m (n = 4). (B) Representative HE staining image of livers from mice intravitreal injected with Kavain or DMSO (Ctrl) (n = 3). Scale bar = 50 μm. *: P < 0.05.

Table S. 1. Information about the antibodies used in this study

Antibodies	Cat no.	Company	Source	Dilution of IF	Dilution of WB
Neutrophil	ab53457	Abcam	Rat	1:400	-
F4/80	ab16911	Abcam	Rat	1:400	-
Ki67	ab156956	Abcam	Rat	1:400	-
HIF-1 α	sc-53546	Santa	Mouse	-	1:1000
VEGF-A	E9X8Q	Cell Signaling Technology	Rabbit	-	1:1000
p-VEGFR2	#2478	Cell Signaling Technology	Rabbit	-	1:1000

VEGFR2	#2479	Cell Signaling Technology	Rabbit	-	1:1000
β-actin	81115-1-RR	Proteintech	Rabbit	-	1:1000
